# *Acanthopanax senticosus* Polysaccharide Enhances the Pathogen Resistance of Radiation-Damaged *Caenorhabditis elegans* through Intestinal p38 MAPK-SKN-1/ATF-7 Pathway and Stress Response

**DOI:** 10.3390/ijms23095034

**Published:** 2022-05-01

**Authors:** Mengyao Liu, Nana Li, Shan Shan, Yudong Shi, Yuanbing Zhu, Weihong Lu

**Affiliations:** 1 Institute of Extreme Environment Nutrition and Protection, Harbin Institute of Technology, Harbin 150001, China; liumengyao621@gmail.com (M.L.); 21S025145@stu.hit.edu.cn (N.L.); 15B925035@hit.edu.cn (S.S.); 19B925085@stu.hit.edu.cn (Y.Z.); 2 National and Local Joint Engineering Laboratory for Synthesis, Transformation and Separation of Extreme Environmental Nutrients, Harbin Institute of Technology, Harbin 150001, China; 3 School of Chemical Engineering and Chemistry, Harbin Institute of Technology, Harbin 150001, China; as-king@163.com

**Keywords:** *Acanthopanax senticosus* polysaccharide, radiation, immune, p38 MAPK, stress response, *Caenorhabditis elegans*

## Abstract

With the advancement of science and technology, humans are chronically exposed to ionizing radiation. It is crucial to look for efficient and low-toxic anti-radiation agents. Through preliminary screening, we found that *Acanthopanax senticosus* polysaccharide (ASPS) played a major role in regulating immune damage caused by radiation. The objective of this study was to apply the *Caenorhabditis elegans*—*P. aeruginosa* (PA14) infection model to illuminate the mechanism of ASPS increasing the pathogen resistance of radiation-damaged nematodes. Results indicated that ASPS (1 mg/mL) significantly enhanced the pathogen resistance of radiation-damaged nematodes by directly elevating the immune response of nematodes rather than by affecting the bacterial activity. Through further research on the p38 MAPK signaling pathway and related mutants, we found that ASPS functioned by the p38 MAPK pathway in the intestine, and SKN-1, ATF-7 as the downstream targets of PMK-1 participated the regulation of ASPS. In addition, ASPS markedly alleviated the stress status of damaged nematodes by regulating oxidative stress. Collectively, our findings suggest that ASPS enhances the pathogen resistance of radiation-damaged nematodes through the intestinal p38MAPK-SKN-1/ATF-7 pathway and stress response.

## 1. Introduction

With the development of science and technology, more and more radiation from different sources affects human health, such as space radiation, nuclear pollution, and medical radiation. Radiation can cause complex biological and chemical changes in the human body, resulting in damage to the function and structure of cells, tissues, and life systems [1]. The immune system is extremely sensitive to ionizing radiation. Radiation can reduce the number of immunocompetent cells, dysregulate cytokine networks, suppress immune function, and increase the chance of infection with bacteria and viruses [2]. Therefore, actively preventing and protecting against the immune damage caused by radiation has become a focal issue.

*Acanthopanax senticosus*, a medicinal and food homologous plant, is distributed in China, Japan, and North Korea. The extract of *A. senticosus* mainly contains polysaccharides, flavone, and saponin. It is reported that *A. senticosus* has an excellent anti-radiation effect and related research has focused on its protective effect on radiation-induced nerve injury [3,4]. However, it has not been reported whether the functional components in *A. senticosus* can protect against radiation-induced immunity decline. Therefore, we investigated the regulatory effects of polysaccharides, flavonoids, and saponins in *A. senticosus* on radiation-damaged immune systems. Through preliminary screening, we found that *A. senticosus* polysaccharide (ASPS) played a major role in regulating immune damage caused by radiation. The structure of ASPS has been analyzed, the skeletal structure is composed of HG smooth area and RG hairy area, HG is repeated → ^4^GalA^1^ →, RG is repeated → ^4^GalA^1^ → ^2^Rhap^1^ →. The side chain is mainly composed of galactan, arabinan, and arabinogalactan [5]. In this study, we explored the specific function and mechanism of ASPS in regulating immune damage caused by radiation.

*C. elegans* has been widely utilized owing to its advantages of easy cultivation, easy observation, plentiful mutant strains, and highly conserved pathways. *Pseudomonas aeruginosa* (PA14), one of the most typical bacterial pathogens, can cause lethal infection in *C. elegans* by secreting virulence factors, including protease, elastase, biofilm, etc. [6]. *C. elegans*—PA14 infection model is currently a novel and excellent model in studying immune-boosting drugs [7]. Researchers have found several immune response genes and signal pathways in nematodes that are crucial for innate immune regulation, including early response genes: IRG-1, HSF-1 and C29F3.7 [8], late response genes: LYS-1, SPP-1, and ABF-1 [9], p38MAPK/PMK-1 [10] signal pathway. In *C. elegans*, p38 MAPK pathway contains mitogen-activated protein kinase (MAPK) encoded by *pmk-1* gene, MAPK kinase (MAPKK) encoded by *sek-1* gene and MAPK kinase kinase (MAPKKK) encoded by *nsy-1* gene. *atf-7* and *skn-1*, encoding basic region-leucine zipper (bZIP) transcription factors, can serve as the downstream targets of pmk-1 to regulate the immune process and stress resistance [11]. In addition, there is a significant relationship between changes in immune function and stress response. Changes or accumulation of extracellular reactive oxygen species (ROS) will alter the immune response, resulting in a systemic inflammatory state and increased susceptibility to disease [12]. Canonical oxidative stress genes [13,14,15] have been thoroughly studied in *C. elegans*.

Therefore, we utilized the *C. elegans*–PA14 infection model to illuminate the property and mechanism of ASPS in regulating the immune damage caused by radiation. Our findings suggest that ASPS enhances the pathogen resistance of radiation-damaged nematodes through the intestinal p38 MAPK –SKN-1/ATF-7 pathway and stress response.

## 2. Results

### 2.1. ASPS Enhanced the Pathogen Resistance of Radiation-Damaged Nematodes

**Figure 1:**
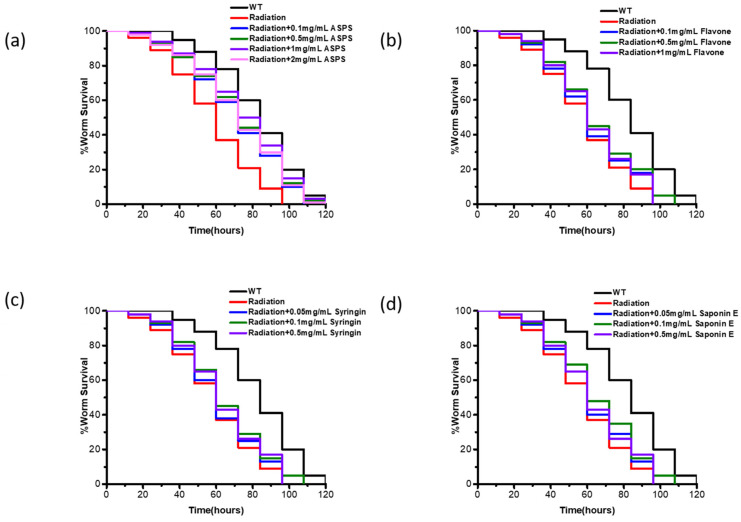
ASPS enhanced the pathogen resistance of radiation-damaged nematodes. (**a**–**d**) Survival curve of PA14-infected nematodes cultured with different concentrations of ASPS, flavonoids, syringin, saponin E. 1mg/mL ASPS could significantly improve the survival of radiation-damaged nematodes. n = 3 biological replicates. *p* < 0.01 (log-rank test).

To determine which active compound in *A. senticosus* played a major role in improving pathogen resistance of radiation-damaged nematodes, we chose *C. elegans*-PA14 infection model to measure the protective effect of ASPS, flavone, syringin, and saponin E, respectively. Results indicated that radiation markedly decreased the survival time of nematodes under PA14 infection (Figure 1). Flavone, syringin, and saponin E had no obvious protective effect (Figure 1b–d). However, 1 mg/mL ASPS could significantly improve the survival rate of radiation-damaged nematodes under PA14 infection (Figure 1a, *p* < 0.01). The mean lifespan of radiation-damaged nematodes under PA14 infection increased from 58.20 ± 2.22 to 75.00 ± 2.57 h after adding 1 mg/mL ASPS. (Table S2). Continuing to increase the concentration of ASPS did not increase the protective effect.

### 2.2. ASPS Did Not Cause Toxic Effects on Radiation-Damaged C. elegans

**Figure 2:**
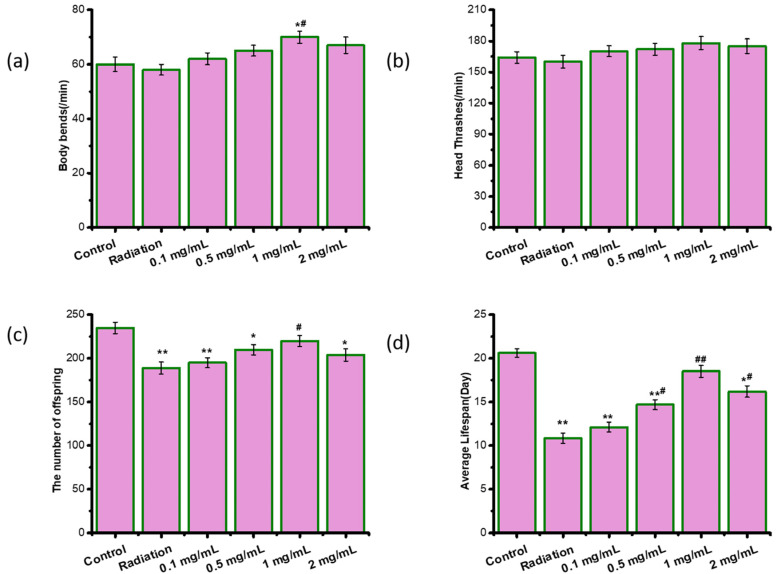
ASPS did not cause any toxic effects on radiation-damaged *C. elegans*. (**a**) The body bends. n = 20. (**b**) The head thrashes. n = 20. (**c**) The number of offspring. n = 20. (**d**) The average lifespan. n = 3. Significant difference from the control group was designated as * *p* < 0.05 and ** *p* < 0.01, and significant difference from the radiation group was designated as ^#^ *p* < 0.05 and ^##^ *p* < 0.01, respectively.

To evaluate whether ASPS caused toxic and side effects, we measured the movement (body bends and head thrashes), the number of offspring and the lifespan of radiation-damaged nematodes. We found that radiation did not cause damage to the movement, but significantly reduced the number of offspring and the lifespan (Figure 2). 1mg/mL of ASPS significantly increased the body bends, the number of offspring and the lifespan of radiation-damaged nematodes (Figure 2). ASPS did not show side effects or toxicity on *C. elegans* within the experimental concentration range. What’s more, ASPS at 1 mg/mL protected radiation-damaged nematodes better than other doses tested. Therefore, ASPS was added at the concentration of 1 mg/mL in NGM plates for subsequent experiments**.**

### 2.3. ASPS Protected Radiation-Damaged Nematodes against PA14 by Elevating Innate Immune Response

**Figure 3:**
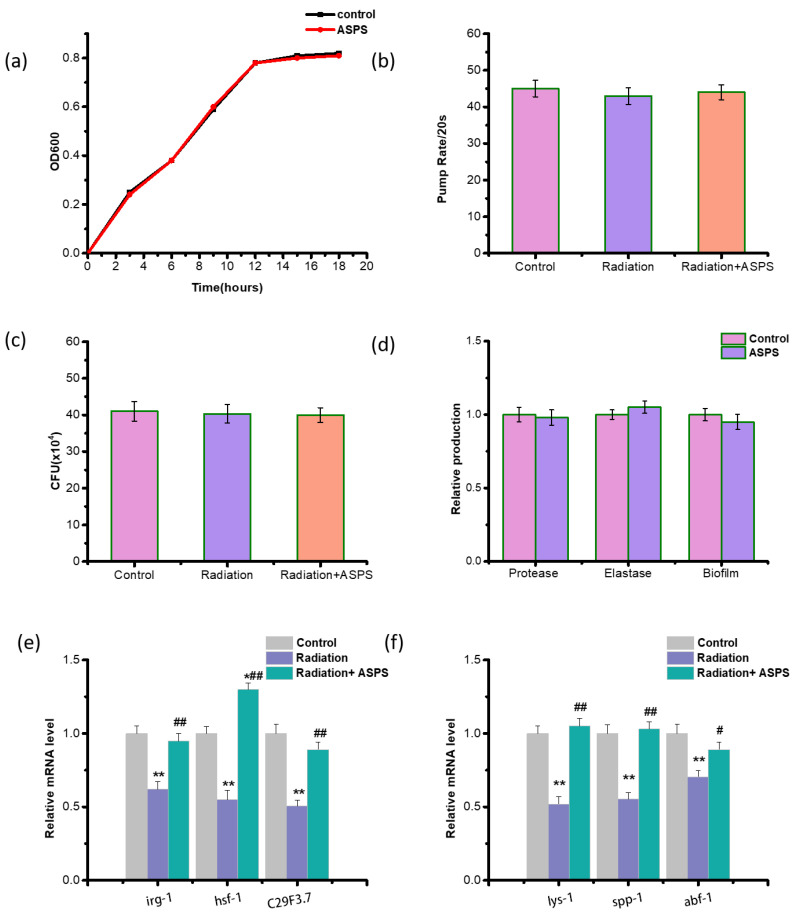
ASPS protected radiation-damaged nematodes against PA14 by elevating innate immune response. (**a**) PA14 growth curve in the presence of 1mg/mL ASPS. n = 3. (**b**) Pharynx pump rate of nematodes at 24 h after PA14 infection. n = 20. (**c**) Colony-forming units (CFUs) of PA14 in nematodes at 24 h after PA14 infection. n = 3. (**d**) PA14 virulence factors (protease activity, elastase activity and biofilm formation). PA14 was cultured at 37 °C for 24 h with or without ASPS. n = 3. (**e**) Early immune response genes. The genes were measured at 12 h after PA14 infection. n = 3. (**f**) Late immune response genes. The genes were measured at 24 h after PA14 infection. n = 3. Significant difference from the control group was designated as * *p* < 0.05 and ** *p* < 0.01, and significant difference from the radiation group was designated as ^#^ *p* < 0.05 and ^##^ *p* < 0.01, respectively.

Several studies have suggested that the protective mechanism of active substances involves inhibiting PA14 growth, intake, colonization, virulence factors, and directly enhancing the immune response of nematodes [16]. To identify the specific mechanism of ASPS, we first examined PA14 growth curve, virulence factors (protease activity, elastase activity, and biofilm formation), pharynx pump rate of nematodes, and the content of PA14 in nematodes at 24 h after infection. Results exhibited radiation and ASPS did not affect all these factors, meaning that ASPS and radiation did not work by affecting PA14 (Figure 3a–d). Then we evaluated three early immune response genes (*irg-1*, *hsf-1*, C29F3.7) and three late immune response genes (*lys-1*, *spp-1*, *abf-1*) of nematodes. We found that radiation significantly downregulated both early and late response genes and resulted in immunosuppression in nematodes, indicating that radiation had broad effects on the immune system of nematodes. However, ASPS significantly upregulated both early and late response genes, suggesting that ASPS might have multiple targets and protected radiation-damaged nematodes against PA14 by directly elevating innate immune response (Figure 3e–f).

### 2.4. ASPS Promoted Innate Immunity of Radiation-Damaged Nematodes Through Intestinal p38 MAPK Pathway

**Figure 4:**
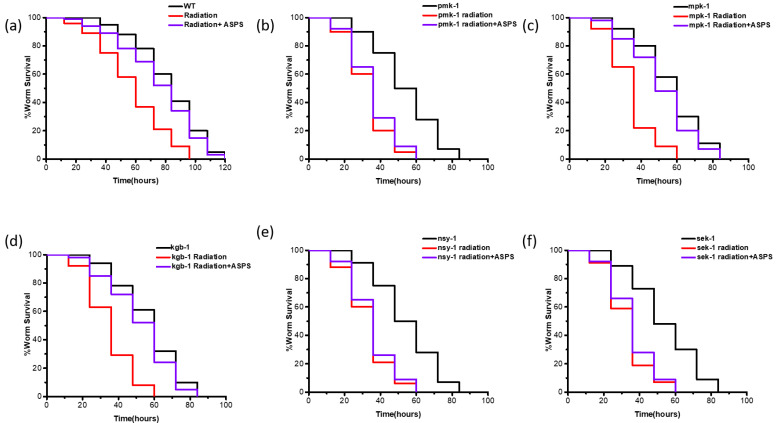
ASPS promoted innate immunity through p38 MAPK pathway. (**a**–**f**) Survival curve of PA14-infected mutants relating to immune signaling pathway. Mutants of core components in the p38 signaling pathway (*nsy-1 sek-1 pmk-1*) failed to increase survival rate after adding ASPS. n = 3. (log-rank test).

To investigate the molecular mechanism of ASPS enhancing the immune response of radiation-damaged nematodes, we screened several immune-related signaling pathways, including p38 MAPK/PMK-1, ERK-MAPK/MPK-1, and JNK-MAPK/KGB-1. We used nematode mutants related to these signaling pathways to conduct PA14 infection experiments. Radiation reduced the survival ability of mutant nematodes. Interestingly, ASPS failed to increase the survival time of radiation-damaged *pmk-1* mutant, but it still significantly augmented the survival time of radiation-damaged *mpk-1* and *kgb-1* mutants under PA14 infection (Figure 4b–d, Table S2). The p38 MAPK pathway is mediated by the NSY-1→SEK-1→PMK-1 cascade reaction, we further examined the core components of the p38 signaling pathway, *nsy-1* and *sek-1*. Similarly, ASPS could not influence the survival of radiation-damaged *nsy-1* and *sek-1* mutants under PA14 infection (Figure 4e,f, Table S2). Therefore, ASPS mainly affected the resistance of radiation-damaged nematodes to PA14 through the p38 MAPK signaling pathway.

**Figure 5:**
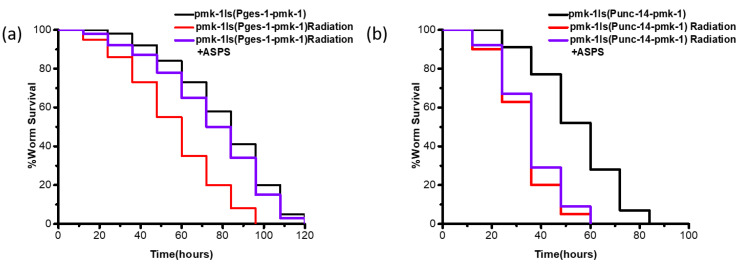
ASPS functioned in the intestine. (**a**,**b**) Tissue-specific rescue experiments. ASPS could significantly enhance the immune capacity of mutant expressing pmk-1 only in the intestine. n = 3. *p* < 0.01 (log-rank test).

In *C. elegans*, *pmk-1* is expressed mainly in intestinal cells and head neurons [17]. In order to explore the tissue-specific activity of ASPS, we performed tissue-specific rescue experiments, expressing *pmk-1* only in the intestine by intestine-specific promoter *ges-1* or in nerve by nerve-specific promoter *unc-14*, and then conducted PA14 infection experiments. ASPS could significantly increase the survival of mutant expressing *pmk-1* only in the intestine (Figure 5a, Table S2) (*p* < 0.01), but it failed to improve the survival of mutant expressing *pmk-1* only in nerve (Figure 5b). At the same time, we performed *sek-1* and *nsy-1* tissue-specific rescue experiments, and the results were consistent with *pmk-1*(results are not shown). Taken together, ASPS played a role through intestinal p38 MAPK signaling pathway.

### 2.5. ASPS Affected Downstream Genes of p38 MAPK Signaling Pathway

**Figure 6:**
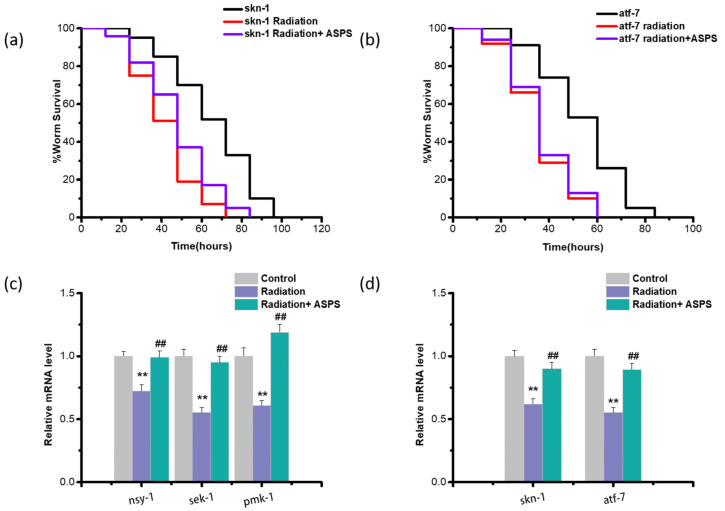
ASPS affected the genes related to p38 MAPK signaling pathway. (**a**,**b**) Survival curve of PA14-infected *skn-1* mutant and *atf-7* mutant. (**c**,**d**) The expression of genes encoding p38 MAPK pathway and *pmk-1* targeted genes. n = 3. Significant difference from the control group was designated as ** *p* < 0.01, and significant difference from the radiation group was designated as ^##^ *p* < 0.01, respectively.

In *C. elegans*, *atf-7* and *skn-1,* encoding bZIP transcription factors, can serve as the downstream targets of *pmk-1* to regulate the immune process and stress resistance [18]. In order to determine the function of *atf-7* and *skn-1* in the ASPS regulation process, we used *atf-7* mutant and *skn-1* mutant for PA14 infection experiments. ASPS did not markedly enhance the resistance of these two mutants (Figure 6a,b, Table S2), indicating *atf-7* and *skn-1* were critical for ASPS to function. In addition, we measured the expression of genes related to the p38 MAPK pathway. Radiation downregulated the expression of these genes, the addition of ASPS significantly upregulated these genes (Figure 6c,d). Results demonstrated ASPS activated the p38 MAPK-SKN-1/ATF-7 pathway.

### 2.6. ASPS Regulated Oxidative Stress

**Figure 7:**
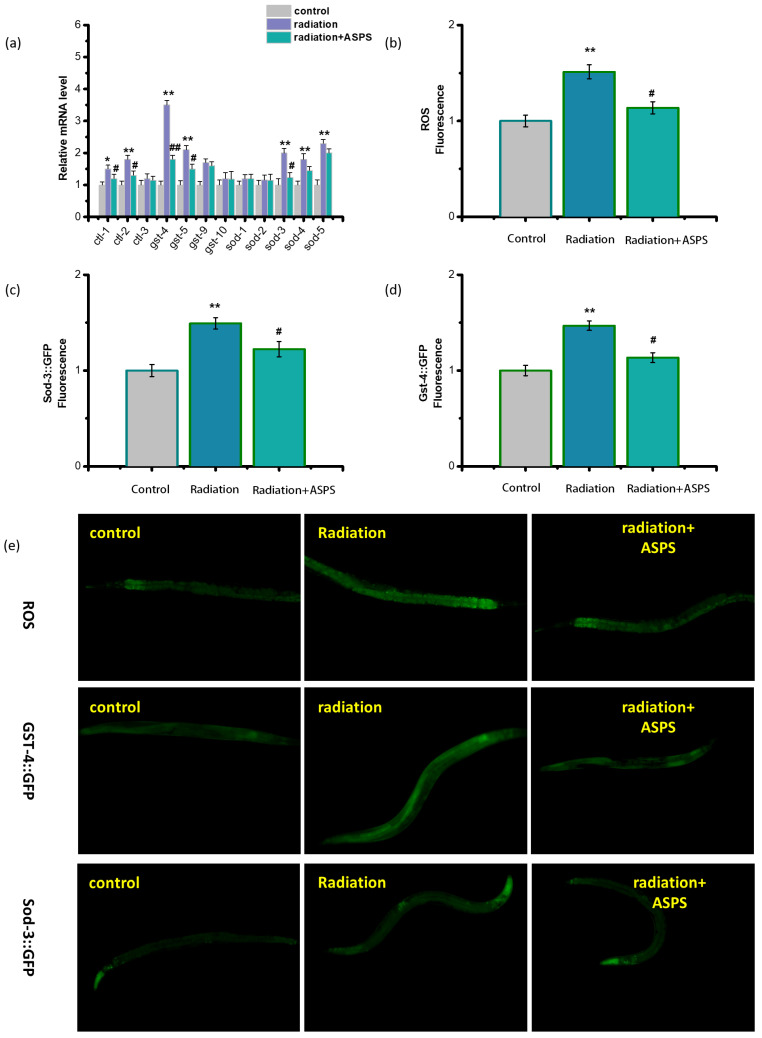
ASPS regulated oxidative stress. (**a**) The expression of canonical oxidative stress genes. n = 3. (**b**–**d**) Quantification of the fluorescence intensity of the microscope pictures. n = 20. (**e**) Fluorescence pictures of ROS, *gst-4*::GFP, *sod-3*::GFP reporter worms. Significant difference from the control group was designated as * *p* < 0.05 and ** *p* < 0.01, and significant difference from the radiation group was designated as ^#^ *p* < 0.05 and ^##^ *p* < 0.01, respectively.

Radiation can indirectly cause oxidative stress. There is a significant relationship between changes in immune function and oxidative stress. In order to explore whether ASPS affected immunity by regulating the oxidative stress of radiation-damaged nematodes, we measured the content of ROS in nematodes and the expression of oxidative stress genes related to the glutathione, catalase and the superoxide dismutase family. As shown in Figure 7a–d, radiation induced obvious oxidative stress in *C. elegans*, the content of ROS increased obviously. ASPS significantly reduced the ROS content and the expression level of most oxidative stress genes of radiation-damaged nematodes, especially *sod-3* and *gst-4*. Superoxide dismutase (SOD) is an antioxidant enzyme in nematodes and the primary substance for free radical scavenging. The expression of SOD is directly related to the level of ROS. *Sod-3* is considered to be the gene with the largest expression change in the SOD gene family. The endogenous products of oxidative damage are highly cytotoxic, including membrane lipid peroxides and DNA oxidative degradation products. Glutathione sulfhydryl transferase (GST) enables glutathione to bind to these endogenous electrophiles to achieve detoxification. *gst-4* can characterize the oxidative stress state. We used transgenic nematodes CF1553 and CL2166 to characterize the expression of SOD-3 and GST-4, respectively. As shown in Figure 7e, ASPS reduced the fluorescence intensity of radiation-damaged transgenic nematodes, which was consistent with the gene expression of *sod-3* and *gst-4*. Results revealed ASPS significantly alleviated the oxidative stress status of radiation-damaged animals.

## 3. Discussion

Through screening the active compound of *A. senticosus*, we discovered that ASPS played a major role in regulating immune damage caused by radiation. Therefore, we further explored the mechanism here. In recent years, *C. elegans* has been proven to be an ideal model for radiation-related research [19,20]. What’s more, it is conducive for in-depth study of signaling pathways [21]. As a result, we utilized the *C. elegans*–PA14 infection model to illuminate the mechanism of ASPS in regulating the immune damage caused by radiation.

We discovered ASPS did not restrain the PA14 bacterial activity and virulence factors, but elevated the immune response genes of nematodes, indicating ASPS directly acts on nematodes. We further gained insight into the mechanism by finding that ASPS protected radiation-damaged nematodes through the p38 MAPK pathway. The p38 MAPK signaling pathway plays an extremely important role in the immune and stress response [22]. External stimuli and internal changes can activate the p38 signal pathway, for example, metformin can increase innate immunity through the p38 MAPK pathway [23], p38 MAP kinase promotes resistance to arsenite-induced lethality, and the p38 MAPK pathway is involved in stress responses against rare earth ions [24]. Remarkably, expressing *pmk-1* in the intestine was sufficient for ASPS to function, indicating ASPS mainly worked in the intestine. *Skn-1*, homologous to human nuclear factor erythroid 2-related factor 2 (Nrf2), is implied to be a crucial regulator of immunity [25]. Losing Nrf2 makes mice vulnerable to viruses and activation of Nrf2 reduces the infection by *Salmonella typhimurium*. Our results also proved that *skn-1* took part in regulating immunity. In addition, we found *atf-7* as downstream target of *pmk-1* participated in the process of ASPS regulating immunity. To our knowledge, it was the first report that ASPS functioned through the intestinal p38 MAPK-SKN-1/ATF-7 pathway.

The antioxidant capacity of ASPS has been confirmed. It exhibits strong DPPH and HO scavenging activities [26]. To investigate whether ASPS affected immune ability by regulating the level of oxidative stress of radiation-damaged nematodes, we measured the content of ROS in nematodes and the expression of oxidative stress genes. We found that ASPS reduced the content of ROS and regulated the expression of sod-3 and gst-4. It is recognized that there is a significant relationship between changes in immune function and oxidative stress. Changes or accumulation of extracellular ROS will alter the immune response, resulting in a systemic inflammatory state and increased susceptibility to disease [12]. In addition, oxidative stress can induce the apoptosis pathway and lead to alteration in the components of the immune system [27]. As a result, ASPS might enhance the immune capacity of radiation-damaged nematodes through the regulation of oxidative stress in part.

Collectively, our findings suggest that ASPS enhances the innate immunity of radiation-damaged nematodes through the intestinal p38MAPK-SKN-1/ATF-7 pathway and stress response.

## 4. Materials and Methods

### 4.1. Nematode Strains and Bacterial

Nematodes were maintained on nematode growth medium (NGM) at 20 °C as described before [28]. Nematode strain N2 was obtained from Dalian Maritime University. Some strains were obtained from the *Caenorhabditis Genetics Center* (CGC), including *pmk-1(km25)*, *nsy-1(ag3)*, *sek-1(ag1)*, *skn-1(zj15)*, *atf-7(tm4392)*, CL2166[*gst-4*::*GFP*] and CF1553[*sod-3*::*GFP*]. *pmk-1(km25)Is*(P*ges-1*-*pmk-1*) and *pmk-1(km25)Is*(P*unc-14*-*pmk-1*) were obtained by gene cloning. *pmk-1* was amplified by PCR and then inserted into the vector with *ges-1* promoter or *unc-14* promoter, respectively. The rescue constructs (20–50 ng/µL) were injected into gonads of *pmk-1(km25)* in the young adult stage. We screened the F1 lines that produced transgene-carrying F2 progenies. The integrated transgene alleles were generated through the mutagenesis process of ultraviolet radiation. Briefly, the exchromosomal array transgene animals in young adult stage were irradiated in UV crosslinker (30 mj). The F1 progenies of irradiated P0 animals were cultured and screened.

*P. aeruginosa* strain PA14 was grown in King’s B broth. *E. coli* strain OP50 was cultured in LB broth. They were grown overnight at 37 °C and then spread on NGM plates.

### 4.2. Preparation of A. senticosus Extract

ASPS were extracted and characterized according to the previous study [5]. Briefly, the high-quality *A. senticosus* leaves were extracted with hot water (90 °C) and precipitated with ethanol at a final concentration of 80%. The crude polysaccharide was purified by DEAE-52 and Sephacryl S100 HR gel chromatography column sequentially. As a result, the purified component ASPS was obtained.

Flavone (90%), syringin (98%), and saponin E (98%) were purchased from Nanjing Xinhou Co., Ltd. (Nanjing, China).

### 4.3. Radiation Treatment and the Usage of Extract

After synchronization, the nematodes in the L3–L4 period were treated with ^60^Co-γ ray radiation, the radiation dose was 50 Gy. Gamma radiation exposure was carried out in a Co-60 irradiation facility of the Maize Research Institute, Heilongjiang Academy of Agricultural Sciences. The radiation dose was determined by potassium dichromate spectrophotometry.

The extract solution was mixed in NGM that were used from 24 h before irradiation until the end of experiment.

### 4.4. PA14 Infection Assay

PA14 infection experiments were performed as before [29]. Briefly, PA14 cultured overnight was evenly spread on NGM plates with or without ASPS, NGM plates were placed in a 37 °C incubator for 24 h, then irradiated adults were placed on them, and the survival status of nematodes was recorded every 12 h. When the nematode did not respond to the touch of the platinum needle, the nematode was considered dead. Experiments were repeated three times. About 100 nematodes were determined in each group.

### 4.5. Pharynx Pump Determination

The measurement of pharynx pump movement (feeding frequency) was based on Lazakovitch et al. [30]. Nematodes capture food from outside by the rhythmic contraction of the pharynx. It reflects the feeding rate. Nematodes were placed on the surface of NGM plates with PA14, the number of pharynx pump movement within 20 s was observed and counted at 24 h after infection. About 20 nematodes were randomly determined in each group.

### 4.6. Colony-Forming Unit (Cfu) Measurement

*C. elegans* infected with PA14 for 24 h were transferred to an M9 buffer containing levamisole to anesthetize nematodes and stop the movement of the pharynx. Then nematodes were placed on a food-free medium for 15 min to further remove the bacteria in vitro and were washed twice with an M9 buffer. They were ground and diluted to the appropriate concentration step by step. Finally, the lysates were spread on LB plates containing gentamicin to screen PA14. After overnight culture at 37 °C, the number of clones was counted. About 20 nematodes were randomly determined in each group.

### 4.7. Protease Activity, Elastase Activity and Biofilm Assays

Protease activity was detected by examining the skim milk hydrolysis effectiveness of the secreted protease [31]. Briefly, PA14 was cultured overnight in KB broth with or without ASPS (1 mg/mL) at 37 °C. Then, 50 µL supernatant of PA14 KB culture was added to 450 µL skim milk (0.5% (*w/v*)) in Tris HCl buffer and the absorbance at OD600 was determined at 24-h incubation.

Elastase activity was examined by elastin-Congo red [32]. A total of 100 μL supernatant was added to 1 mL of 10 mM Na_2_HPO4 and 10 mg of elastin-Congo red and was incubated for 4 h at 37 °C with 180 rpm shaking. Then the tubes were centrifuged at 8000× *g* for 10 min and the absorbance was measured at 495 nm.

The biofilm assay was conducted as described before [33]. The PA14 biofilm was stained with 1% crystal violet, then it was solubilized with 30% acetic acid in water and determined using a microplate reader at 550 nm.

### 4.8. Measurement of ROS

2,7-dichlorodihydrofluorescein diacetate and ageneral molecular probe for ROS measurement in vivo, was used to measure ROS level. Irradiated nematodes were cultured for 24 h. Then they were transferred to the 24-well microtiter plate containing H2DCF-DA (final concentration 50 μM) and incubated at 20 °C for 3 h in the dark. The nematodes were photographed using a Nikon Ti2-U fluorescence microscope (Nikon, Japan). Image-Pro Plus 6.0 was used to measure and analyze relative fluorescence intensity of nematodes. About 20 nematodes were randomly determined in each group.

### 4.9. Microscopic Imaging

Irradiated transgenic adults sod-3::GFP and gst-4::GFP were picked to agar pads and recorded by a Nikon Ti2-U fluorescence microscope. Image-Pro Plus 6.0 was used to measure and analyze relative fluorescence intensity. About 20 nematodes were randomly determined in each group.

### 4.10. Quantitative RT-PCR Assay

Total RNA was extracted with the MiniBEST Universal RNA Extraction Kit. cDNA was obtained using the PrimeScript RT reagent Kit with gDNA Eraser. Real-time PCR was performed using a CFX96TM real-time PCR system (Bio-Rad, CA, USA). The primers used in this study are listed in Table S1.

### 4.11. Statistical Analysis

All results are expressed as the mean ± standard error of the mean. The log-rank test was used to analyze differences in survival rates. Other data analysis was evaluated using one-way analysis of variance (ANOVA) followed by Bonferroni post-hoc tests for multiple comparisons. *p* < 0.05 and *p* < 0.01 were considered statistically significant.

## 5. Conclusions

Using the *C. elegans*–PA14 infection model, we discovered ASPS significantly protected radiation-damaged nematodes from the infection by directly acting on nematodes rather than by affecting the bacterial condition. ASPS functioned through the p38 MAPK pathway in the intestine and *skn-1*, *atf-7* as the downstream targets of *pmk-1* participated the regulation of ASPS. In addition, ASPS significantly alleviated the stress status of damaged nematodes by regulating oxidative stress. It is the first time that the mechanism of *ASPS* to improve the immunity of radiation-damaged nematodes was revealed, laying a solid foundation for its promotion as anti-radiation food.

## Data Availability

Not applicable.

## Supplementary Materials

The following supporting information can be downloaded at: https://www.mdpi.com/article/10.3390/ijms23095034/s1.
