# Supplementary material for: Acanthopanax senticosus Polysaccharide Enhances the Pathogen Resistance of Radiation-Damaged Caenorhabditis elegans through Intestinal p38 MAPK-SKN-1/ATF-7 Pathway and Stress Response
Source: Int J Mol Sci. 2022 May 1;23(9):5034. doi: 10.3390/ijms23095034 (PMC9103771; doi:10.3390/ijms23095034)
Supplement: Supplementary file 1 [file ijms-23-05034-s001.zip › ijms-1676131-supplementary.pdf]

Table S1: The primers

Genes	**Forward primer sequence**	**Reverse primer sequence**
act-1	ACGACGAGTCCGGCCCATCC	GAAAGCTGGTGGTGACGATGGTT
tba-1	TCAACACTGCCATCGCCGCC	TCCAAGCGAGACCAGGCTTCAG
irg-1	AAGCAGCATGCGTATTTTCA	GCAGCTTCTCCTTTTTCTCC
hsf-1	CAGCCAACAGGGAATCAAAT	TGCTGCTCCAGAAACTGAAA
C29F3.7	GATCGGCAACTTTACCTCCA	AATTGTGGCGGATATTCTGG
lys-1	TTCGGATCTTTCAAGAAGGC	GGGATTCCAACAACGTAAA
spp-1	TGAACATCGGAACTCTTTGC	TCAGCTCTTCCTCACACTCG
abf-1	TGCCTTCTCCTTGTTCTCCT	ATCCTCTGCATTACCGGAAC
pmk-1	CGACTCCACGAGAAGGAT	ATATGTACGACGGGCATG
sek-1	TGCTCAACGAGCTAGACG	ATGTTCGACGGTTTCACG
nsy-1	TGCGATGAACTACTACGG	CACCCAAATGACCAAATA
skn-1	AGGCTCAACCTCAGAACATG	TACGAGTAGGCGGTCATTTC
atf-7	CTGGAGAACTTGACGTGGCA	ATCCGACATTGTTCCGGCAT
ctl-1	GAATGTGAAGAATTATTTCGCTGA	AACTCGATTCCTGGGACGAT
ctl-2	CAAGGAACTACTTCGCTGAGG	AATGAGTGTCGGTGTACGAGAA
ctl-3	GAATGTGAAGAATTATTTCGCTGA	AACTCGATTCCTGGGACGAT
gst-4	GATACTTGGCAAGAAAATTTGGAC	TTGATCTACAATTGAATCAGCGTAA
gst-5	GCCAGCATTGAAAGAAACCT	TTTTTCCGTTGAGCTTGAACT
gst-9	CAAGAATTTATTGATGAGCGTATTTCT	ACTTGTCCAAATGGGGTCATA
gst-10	ACTGGAGCAATTATGCGTCA	CTCCCTCGAAGAACATGTCG
sod-1	ACGCTCGTCACGCTTTAC	TCTTCTGCCTTGTCTCCG
sod-2	GGCATCAACTGTCGCTGT	ACAAGTCCAGTTGTTGCC
sod-3	TGACATCACTATTGCGGT	GGGACCATTCCTTCCAAA
sod-4	CACCAGATGACTCGAACA	AATGAGGCAAGAGAGTCG

Table S2: Mean survival time of nematodes under PA14 infection

Figure	strains	treatments	mean survival time (h)	P value (vs radiation group)
Fig1. a	N2	control	82.44±2.16	**
		radiation	58.20±2.22	
		radiation+0.1mg/mL ASPS	70.32±2.51	**
		radiation+0.5mg/mL ASPS	72.00±2.59	**
		radiation+1mg/mL ASPS	75.00±2.57	**
		radiation+2mg/mL ASPS	70.65±2.32	**
Fig1. b	N2	control	82.15±2.13	**
		radiation	57.12±2.18	
		radiation+0.1mg/mL Flavone	59.22±2.10	
		radiation+0.5mg/mL Flavone	60.56±2.05	
		radiation+1mg/mL Flavone	59.72±2.07	
Fig1. c	N2	control	82.79±2.62	**
		radiation	58.34±2.43	
		radiation+0.05mg/mL Syringin	58.96±2.10	
		radiation+0.1mg/mL Syringin	59.87±2.08	
		radiation+0.5mg/mL Syringin	59.63±2.21	
Fig1. d	N2	control	81.94±2.16	**
		radiation	58.34±2.51	
		radiation+0.05mg/mL SaponinE	59.13±2.31	
		radiation+0.1mg/mL SaponinE	60.47±2.26	
		radiation+0.5mg/mL SaponinE	59.78±2.34	
Fig4. a	N2	control	82.44±2.16	**
		radiation	58.20±2.22	
		radiation+1mg/mL ASPS	75.32±2.51	**
Fig4. b	*pmk-1*	control	54.00±1.69	**
		radiation	33.00±1.19	
		radiation+1mg/mL ASPS	35.40±1.28	
Fig4. c	*nsy-1*	control	54.36±1.71	**
		radiation	34.48±1.25	
		radiation+1mg/mL ASPS	35.20±1.34	

Fig4. d	*sek-1*	control	53.72±1.22	**
		radiation	34.63±1.07	
		radiation+1mg/mL ASPS	35.09±1.31	
Fig5. a	*pmk-1Is*(P*ges-1-pmk-1*)	control	80.03±2.08	**
		radiation	57.31±2.17	
		radiation+1mg/mL ASPS	74.38±2.3	**
Fig5. b	*pmk-1Is*(P*unc-14-pmk-1*)	control	54.87±1.16	**
		radiation	34.93±1.09	
		radiation+1mg/mL ASPS	35.62±1.27	
Fig6. a	*skn-1*	control	64.83±1.34	**
		radiation	41.35±1.23	
		radiation+1mg/mL ASPS	45.40±1.34	
Fig6. b	*atf-7*	control	53.89±1.33	**
		radiation	33.63±1.09	
		radiation+1mg/mL ASPS	35.07±1.21	

**p<0.01 (log-rank test)
